# Supplementary figures and images for: Characterizing medaka visual features using a high-throughput optomotor response assay
Source: PLoS One. 2024 Jun 28;19(6):e0302092. doi: 10.1371/journal.pone.0302092 (PMC11213317; doi:10.1371/journal.pone.0302092)

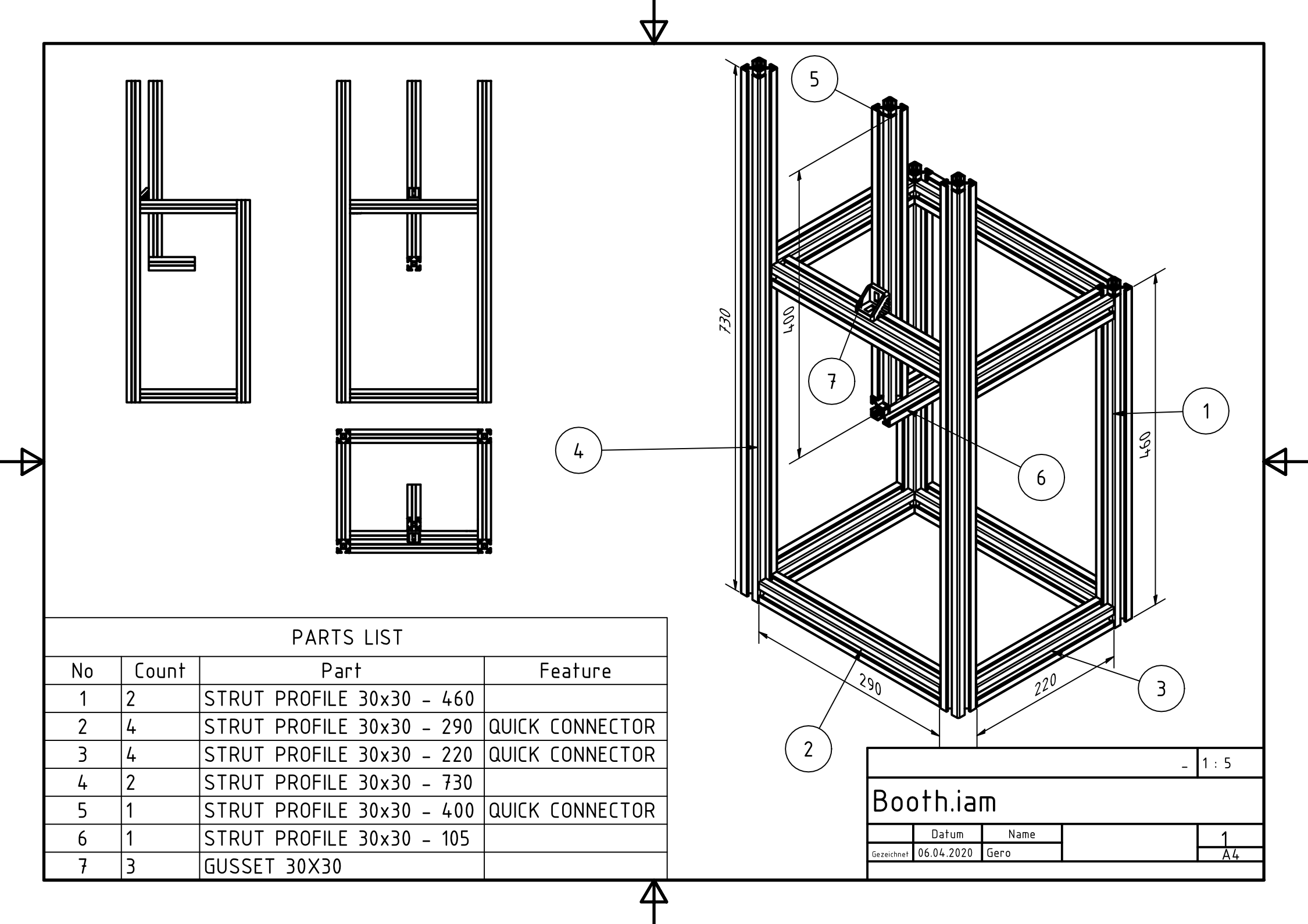

Supplement: S1 Fig — (TIF) [file pone.0302092.s001.tif]

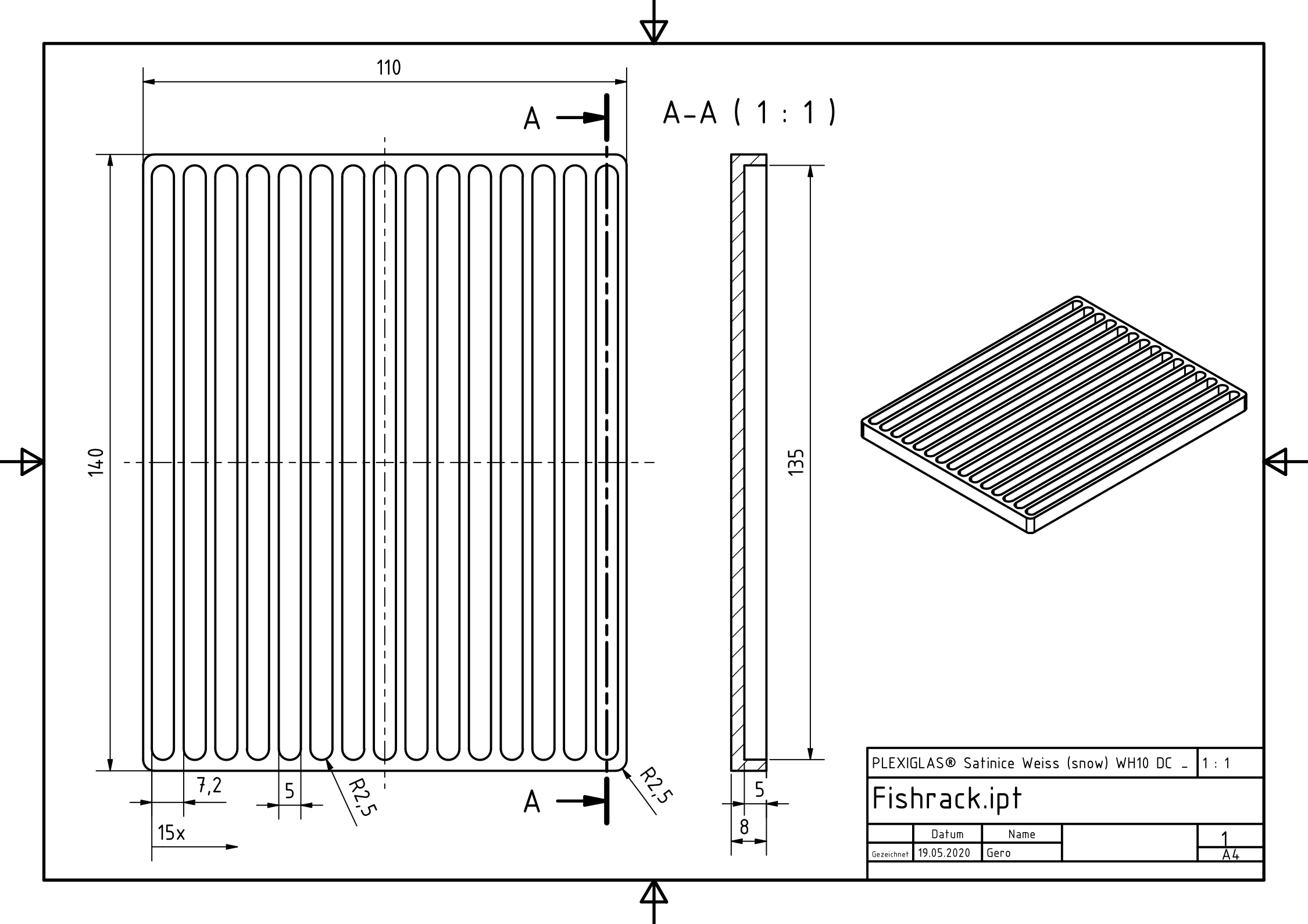

Supplement: S2 Fig — (TIF) [file pone.0302092.s002.tif]

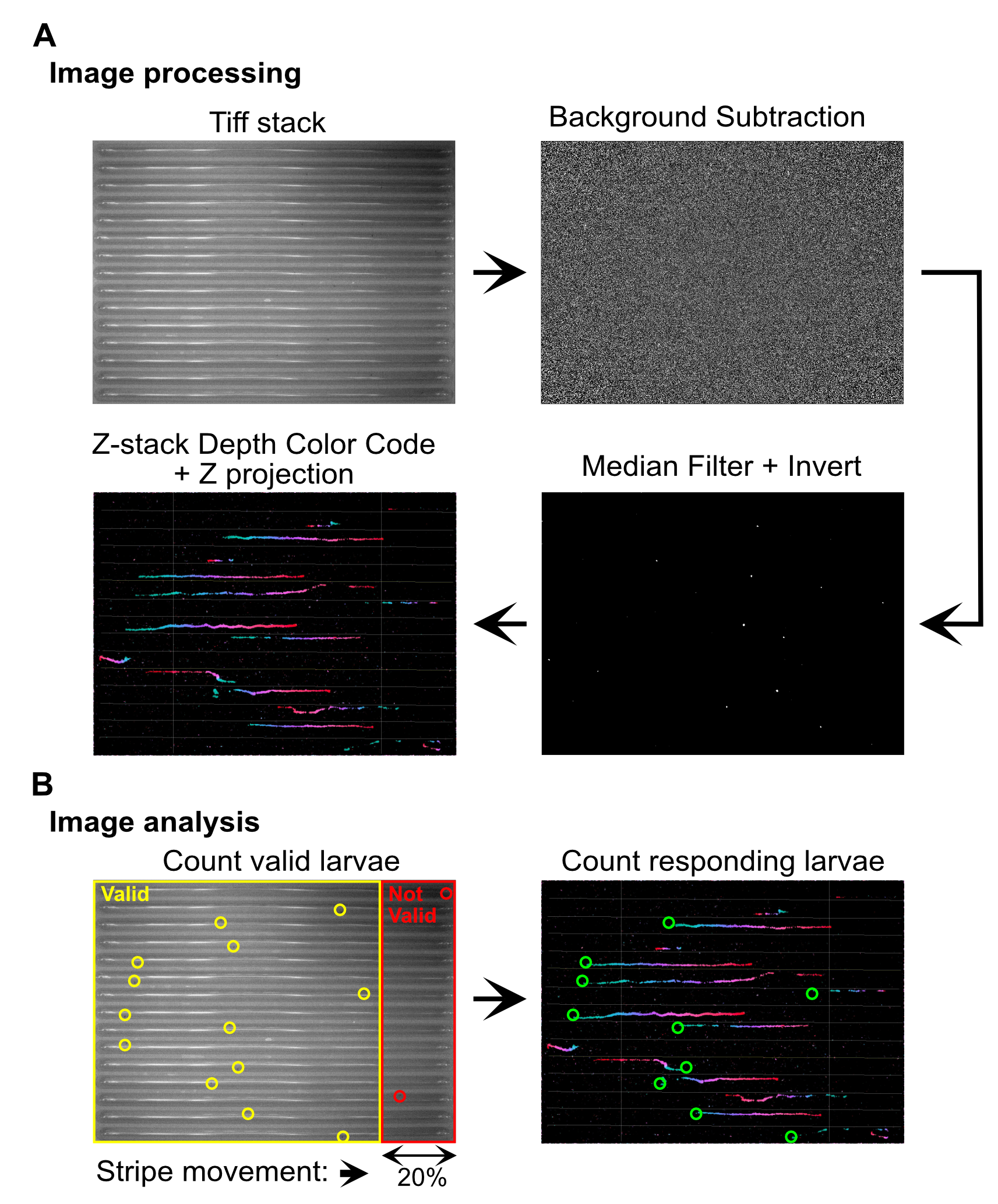

Supplement: S3 Fig — (A) Tiff stack was processed using Fiji as follows. First slice of the stack was duplicated and subtracted from each slice of the stack to subtract the background. Subsequently, noise reduction using median filter and inversion were performed. Lastly, Depth color code plugin and Z projection were used to visualize larval swimming trajectories. (B) For image analysis, the first slice of the stack was used to classify larvae as either valid or non-valid for response rate calculation. Those larvae located more than 27 mm from the lane end (corresponding to 20% of the lane length) were counted as valid (yellow circles). Those larvae located close to the lane end were considered as non-valid (red circles). Z projection images were used to classify larvae as either responsive or non-responsive. Those larvae swimming in the direction of stripe motion for at least 20% of the lane length were considered as responsive (green circles). Arrow indicates direction of stripe movement. (TIF) [file pone.0302092.s003.tif]
